# Retinal angioma of Von hippel-lindau disease: A case report

**DOI:** 10.1016/j.amsu.2022.103292

**Published:** 2022-01-25

**Authors:** O. Nabih, H. Hamdani, L. EL Maaloum, B. Allali, A. EL kettani

**Affiliations:** a Medical Resident at Pediatric Ophthalmology Department, Hopital 20 Aout, 1953, Casablanca, Morocco; b Professor- Pediatric Ophthalmology Department, Hopital 20 Aout, 1953, Casablanca, Morocco; c Professor and Head of Pediatric Ophthalmology Department, Hopital 20 Aout, 1953, Casablanca, Morocco

**Keywords:** Von Hippel-Lindau disease, Retinal angioma, Vascular tumours-Neurofibromatosis, Hemangioma

## Abstract

**Introduction:**

Von Hippel–Lindau disease (VHL), also known as Von Hippel–Lindau syndrome, is a rare genetic disorder with multisystem involvement. It is characterised by the development of multiple vascularised tumours, particularly cerebellar, retinal and/or visceral. The disease can occur at any age and usually starts with retinal hemangioblastomas.

**Case report:**

We report the case of a 45-year-old female patient with no particular pathological history, who. consulted the ophthalmology department for a change of optical correction.

The funds examination showed an uncomplicated bilateral hemangioma with no other associated signs. Fluorescein angiography confirmed the diagnosis by showing in the left eye a multiple retinal hemangioma visible in the mid-periphery facing the branches of the superior temporal arches. The brain MRI showed a multifocal hemangioblastoma in the posterior cerebral fossa. A renal ultrasound returned normal. The patient had undergone photocoagulation of the retinal lesions to avoid any complications.

**Discussion:**

The German ophthalmologist Eugen von Hippel first described angiomas in the eye. The term Von Hippel–Lindau disease was first used in 1936; however, its use became common only in the 1970s.

Tumours called hemangioblastomas are characteristic of von Hippel-Lindau syndrome. These growths are made of newly formed blood vessels and occurs in the periphery of the retina. Spontaneous progression occurs leading to visual impairment as a result of maculopathy or exudative retinal detachment.

Early recognition and treatment of specific manifestations of VHL can substantially decrease complications and improve quality of life.

Conventional treatment of the retinal hemangioblastomas is laser photocoagulation or cryotherapy depending on the location and size of the lesions. It must be based on the patient's visual symptoms and tumor progression.

**Conclusion:**

Management of patients with VHL disease often requires a multidisciplinary approach. The role of the ophthalmologist is important in the management of this condition since the ocular involvement may be indicative of the disease.

## Highlights

• Von Hippel Lindau disease is a systemic condition whose initial signs are usually ocular.
• The ocular lesion is generally one of the major and revealing manifestations of the disease.
• The risk of progression to blindness is important, making early detection and treatment highly beneficial.
• The role of the ophthalmologist is crucial in the management of this condition.

## 1 Introduction

Von Hippel Lindau disease is a multi-systemic, autosomal dominant disorder characterised by the development of multiple vascularised tumours, particularly cerebellar, retinal and/or visceral.

The ocular lesion is generally one of the major and revealing manifestations of the disease, which can permanently compromise the visual prognosis.

We report the case of a 45 year old woman who illustrates the ophthalmological and cerebral manifestations of the condition.

This study has been reported in accordance with the SCARE criteria [1].

## 2 Case report

We describe the case of a 45 year old female patient with no particular pathological history. She consulted the ophthalmology department for a change of optical correction. The patient had a corrected visual acuity of 1.0/1.0.

A confrontation visual field was normal.

**Fig. 1:**
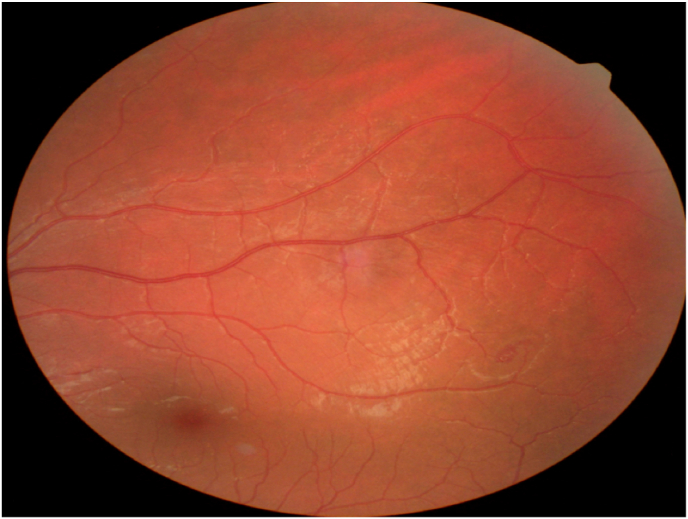
Fundus photograph of the left eye showing a micro angioma in front of a superior temporal retinal artery branch.

The anterior segment was normal. Tonometry was within normal limits. A myosis fundus revealed a normal posterior pole with a suspect vascular lesion over a branch of the superior temporal vascular arch of the right eye. The funds examination after pupillary dilation showed an uncomplicated bilateral hemangioma with no other associated signs. Fig. 1.

Fluorescein angiography confirmed the diagnosis by showing in the left eye a multiple retinal hemangioma visible in the mid-periphery facing the branches of the superior temporal arches.

**Fig. 2:**
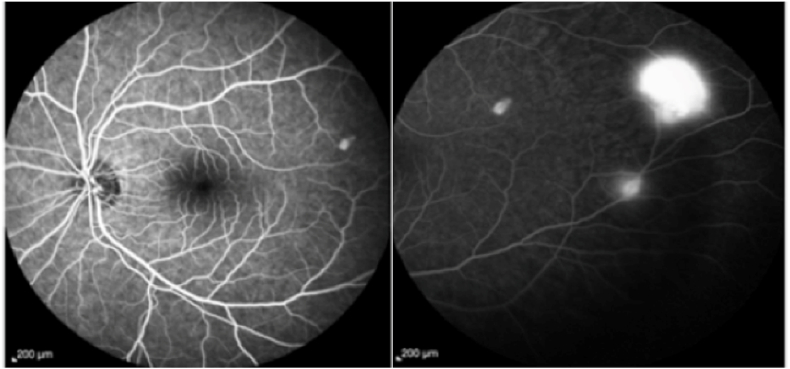
Fluoroscein angiography image at the arteriovenous phase showing vascular diffusions opposite the retinal arterial capillaries.

In the right eye there was a single angioma over the inferior temporal arch branches. Fig. 2.

The suspicion of von hippel lindau disease prompted us to request further investigations.

**Fig. 3:**
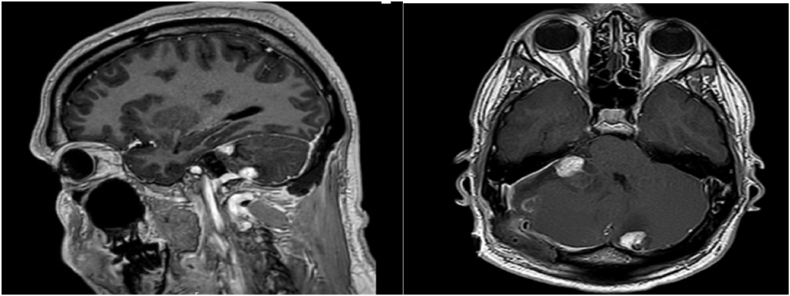
Orbital-brain MRI reveals multiple, non-expanding, centimetric nodular signal abnormalities of the subcortical white matter unaltered after contrast injection.

A brain MRI showed a multifocal hemangioblastoma in the posterior cerebral fossa. Fig. 3.

A renal ultrasound returned normal. A urinary vanylmandelic acid (VMA) assay was normal. Genetic and familial investigation could not be performed due to lack of resources.

VHL disease was considered due to the association of ophthalmic involvement and cerebral hemangioblastomas.

The patient had undergone photocoagulation of the retinal lesions to avoid any complications.

The evolution was marked by a stability of the lesions and a preservation of the visual prognosis during the whole follow-up period (3 years).

## 3 Discussion

The ocular involvement of VHL disease affects the retinal capillaries, also called hemangiomas or hemangioblastomas. The first descriptions of this vascular retinal tumour by Eugen von Hippel were made between 1867 and 1939 [2]. The description was more detailed on the vascular nature of the disease, and since then the term retinal angiomatosis was adopted [[3], [4], [5]]. The genetic nature of the disease was first described by Collins [6,7]. Other publications had described partial and atypical aspects of these vascular tumours [8,9]. In reference to Eugen von Hippel, retinal capillary angioma has been named von Hippel's tumour. Ocular involvement is most often the first sign of the disease. It usually occurs in the third decade, rarely in early childhood [10,11].

Retinal angiomatosis occurs in the periphery of the retina in the form of a tumour with enlarged and tortuous feeder vessels. This vascular architecture is caused by the presence of astrocytes with large lipid-filled vacuoles in the vascular walls separating the vascular channels [12]. Juxta papillary involvement has also been described [13,14]. The early stages of ocular involvement present as microangiomas without vascular enlargement. Spontaneous progression occurs leading to visual impairment as a result of maculopathy [15] or exudative retinal detachment. Fluorescein angiography is an important diagnostic tool for identifying retinal angiomas. Clinically, patients with retinal angiomas show progressive decrease in visual acuity or visual field loss depending on the location of the damage.

The risk of progression to blindness is great in patients with symptomatic retinal angiomatosis, making early detection and treatment highly beneficial [[16], [17], [18]]. The diagnosis is strongly suspected or even confirmed by the fundus aspect of the tumours. Fluorescein angiography remains the gold standard for identifying small angiomas, juxta papillary angiomas or angiomas obscured by epiretinal membranes. MRI or cerebral CT is useful in the detection of cerebral hemangioblastomas, which are most often associated with ocular involvement. The basic treatment for small peripheral retinal angiomas is laser photocoagulation [19,20]. Larger lesions can be treated with cryotherapy or brachytherapy [21,22].

## 4 Conclusion

Von Hippel Lindau disease is a systemic condition whose initial signs are usually ocular and can compromise visual prognosis, making the role of the ophthalmologist crucial in the management of this condition.

## Sources of funding

This study did not receive any sources of funding.

## Author contributions

O.Nabih: drafting the article, study concept, writing the article.

H.Hamdani: acquisition of data.

L.El maaloum: study design.

B.Allali: study concept, revising the article.

A. El kettani: final approval.

## Ethical approval

This type of study does not require any ethical approval by our institution.

## Registration of research studies

1. Name of the registry:

2. Unique identifying number or registration ID:

3. Hyperlink to your specific registration (must be publicly accessible and will be checked):

## Consent

Patient provided written, retrospective consent for publication following detailed explanation of the purpose of manuscript and understanding that no identifiable information was going to be released.

## Guarantor

O.Nabih.

## Provenance and peer review

Not commissioned, externally peer-reviewed.

## Declaration of competing interest

The authors declare no conflict of interest.
